# Shaping the Microstructure of High-Aluminum Cast Iron in Terms of the Phenomenon of Spontaneous Decomposition Generated by the Presence of Aluminum Carbide

**DOI:** 10.3390/ma14205993

**Published:** 2021-10-12

**Authors:** Robert Gilewski, Dariusz Kopyciński, Edward Guzik, Andrzej Szczęsny

**Affiliations:** 1 Faculty of Foundry Engineering, AGH University of Science and Technology, Al. A. Mickiewicza 30, 30-059 Kraków, Poland; gilbert-rg@o2.pl (R.G.); guz@agh.edu.pl (E.G.); ascn@agh.edu.pl (A.S.); 2 Electrical School Complex No. 1, ul. Kamienskiego 49, 30-644 Krakow, Poland

**Keywords:** high-aluminum cast iron, Al_4_C_3_ carbide, spontaneous disintegration of the casting structure

## Abstract

A suitable aluminum additive in cast iron makes it resistant to heat in a variety of environments and increases the abrasion resistance of the cast iron. It should be noted that high-aluminum cast iron has the potential to become an important eco-material. The basic elements from which it is made—iron, aluminum and a small amount of carbon—are inexpensive components. This material can be made from contaminated aluminum scrap, which is increasingly found in metallurgical scrap. The idea is to produce iron castings with the highest possible proportion of aluminum. Such castings are heat-resistant and have good abrasive properties. The only problem to be solved is to prevent the activation of the phenomenon of spontaneous decomposition. This phenomenon is related to the Al_4_C_3_ hygroscopic aluminum carbide present in the structure of cast iron. Previous attempts to determine the causes of spontaneous disintegration by various researchers do not describe them comprehensively. In this article, the mechanism of the spontaneous disintegration of high-aluminum cast iron castings is defined. The main factor is the large relative geometric dimensions of Al_4_C_3_ carbide. In addition, methods for counteracting the phenomenon of spontaneous decay are developed, which is the main goal of the research. It is found that a reduction in the size of the Al_4_C_3_ carbide or its removal lead to the disappearance of the self-disintegration effect of high-aluminum cast iron. For this purpose, an increased cooling rate of the casting is used, as well as the addition of elements (Ti, B and Bi) to cast iron, supported in some cases by heat treatment. The tests are conducted on the cast iron with the addition of 34–36% mass aluminum. The molten metal is superheated to 1540 °C and then the cast iron samples are cast at 1420 °C. A molding sand with bentonite is used to produce casting molds.

## 1. Introduction

Increasing the aluminum content in cast iron can reduce its density and improve its oxidation resistance and abrasion resistance. This type of cast iron can be produced from recycled materials [1,2,3,4,5]. High-aluminum cast iron containing 9–19% by mass Al belongs to the group of white cast iron. In the structure of this cast iron, the ε carbide is shaped, i.e., Fe_3_AlC_x_ and the ferritic metal matrix. High-aluminum cast iron is characterized by good oxidation resistance and creep resistance. It can work in an oxidizing atmosphere up to the temperature of 920 °C, and in an atmosphere containing sulfur compounds up to the temperature of 800 °C. The structure of aluminum cast iron changes significantly in the range of aluminum content of 19–25% by mass. A barrier to the application of this material is the formation of hygroscopic Al_4_C_3_ carbide [2,3,4,5,6,7,8]. This carbide’s properties, and its morphology in the form of needles and plates with a thickness of several micrometers, makes the reaction with water (in liquid or gas form) intense and leads to the destruction of the material, so-called self-decay, as a result of volume expansion after the reaction of aluminum carbide with water particles, according to the reaction:

Al_4_C_3_ + 12H_2_O → 4Al(OH)_3_ + 3CH_4_ ↑, (1)

4Al(OH)_3_ → 2Al_2_O_3_ + 6H_2_O, (2)

Research on Fe–Al–C alloys has shown that the process of their decomposition in the air starts from the surface layers and progresses deeper into the material [2,3,4,5,8]. The reason for this is the formation of the Al(OH)_3_ compound, the volume of which is approximately 2.5 times greater than the specific volume of Al_4_C_3_ carbides.

Another considered process of the decomposition of high-aluminum Fe–Al–C alloys may be the issue of weakening the cohesion of the metal matrix, which occurs as a result of the interaction of hydrogen dissolved in cast iron (the phenomenon of hydrogen embrittlement), which is transferred from the atmosphere during the metallurgical process of the Fe–Al–C alloy, according to the reaction:

2Al + 3H_2_O = Al_2_O_3_ + 3H_2_ (3)

The phenomenon is also known in aluminum composites with carbon reinforcement in the form of fibers and particles, in which the Al_4_C_3_ carbide appears at the stage of component production. It should also be mentioned that similar effects occur in the case of reinforcements using SiC silicon carbide, where an excessively high temperature also affects the structure of the aluminum composite. The formation of aluminum carbide can be found in magnesium composites containing aluminum, reinforced with fibers or carbon particles [9]. The destructive effect of Al_4_C_3_ on the microstructure of materials depends on the amount and the manner of its distribution in the structure of a given material. If there is no continuity of carbide precipitation on the microscale, the adverse effects of the material properties resulting from its reaction with water are negligible and concern only the surface of the product. Thus, it is necessary to control the volume fraction and the morphology of Al_4_C_3_ in cast iron in order to produce a stable, high-aluminum cast iron with a wide range of applications. This phenomenon causes high stress and breaks the continuity of the alloy matrix.

If the phenomenon of the self-decay of high-aluminum cast iron is eliminated, a good and inexpensive material from the Fe-Al intermetallic group [10,11,12,13] could be created. To date, the method of minimizing or completely preventing the formation of the undesirable Al_4_C_3_ phase in high-aluminum cast iron composites (>19% Al) has been investigated [1,5,11]. The absence of aluminum carbide in these castings significantly improves the mechanical strength.

The hypothesis was adopted in this paper that the phenomenon of high-aluminum cast iron self-decay, caused by the presence of aluminum carbide Al_4_C_3_, can be eliminated by changing the morphology of Al_4_C_3_ carbide precipitates.

## 2. Development of the Fe–Al Alloy Phase Equilibrium System and the Fe–Al–C Triple System in the Thermo-Calc Program

**Figure 1:**
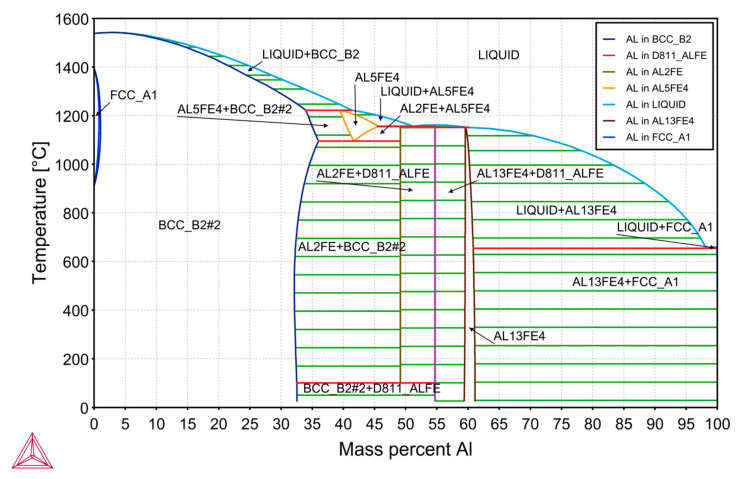
Calculated equilibrium system of Al–Fe alloys obtained in the Thermo-Calc program.

Phase equilibrium diagrams of Fe-Al alloys available in the literature were developed several dozen years ago. In the research, it was decided to redevelop the phase equilibrium diagram of the Fe-Al system, using numerical analysis, based on the Thermo-Calc program. The phase equilibrium diagram of the Fe-Al alloy is shown in Figure 1. It is noteworthy that this diagram shows an additional phase transformation of the alloy within the range of 33–49% mass of Al content, at a temperature of approximately 100–120 °C. This is not visible in the graphs available in the literature related to this field. During this transformation, the crystal lattice is rebuilt—the parameters of the crystal lattice change and this may also cause additional stresses that destroy the internal structure of the alloy. Results were obtained using Thermo-CALC Software version 2019b with thermodynamic database TCFE7 and with the assumption of equilibrium calculations (Figure 1).

**Figure 2:**
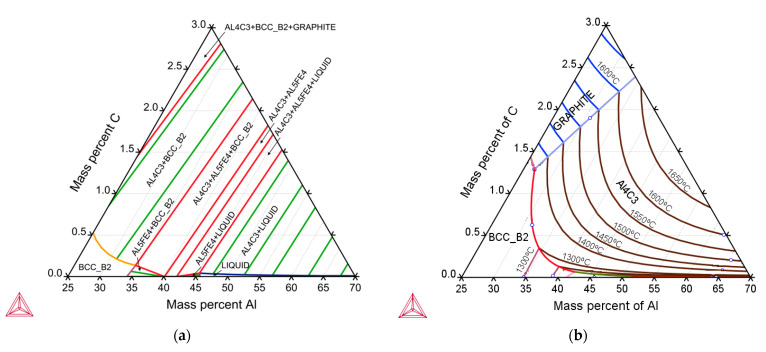
Fe–Al–C equilibrium phase diagram alloys obtained in the Thermo-Calc program at a temperature of 1400 °C (**a**) and determination of the liquidus temperature (**b**).

The Thermo-Calc program was also used to develop the Fe-Al-C triple system. This diagram was produced for a temperature of 1400 °C and is shown in Figure 2a. It is worth noting that, at this temperature, it is possible for graphite formation to start from the carbon content of 2.5% in the alloy. Figure 2b shows the course of the liquidus line in the Fe-Al-C alloy.

## 3. Methodology

A medium-frequency induction furnace with a thyristor inverter, with a capacity of 30 kg, was used to carry out the melts. The molten metal was superheated to 1540 °C and then cast iron samples were cast at 1420 °C. A molding sand with bentonite was used to make casting molds; it included quartz sand as the matrix, bentonite as a binder and water and coal dust as additives. The molds were dried at a temperature of 200 °C. One mold contained three test bars that were 30 mm in diameter and 260 mm in length.

The microscopic observations were made on a Leica optical microscope (MEF4M, Leica Microsystems, Wetzlar, Germany) using the Leica Q Win program (software version 6). The SEM microstructure and the phase composition analysis in the structure of the investigated cast iron, as well as the chemical composition analysis, were carried out on a JEOL 500LV scanning microscope (JEOL Ltd., Tokyo, Japan) with an X-ray microanalysis (EDS) attachment.

## 4. Determining the Hypothesis Concerning the Cause of the Phenomenon of Spontaneous Decay

In order to test the decomposition of high-aluminum cast iron, samples were cast with the chemical composition presented in Table 1.

**Figure 3:**
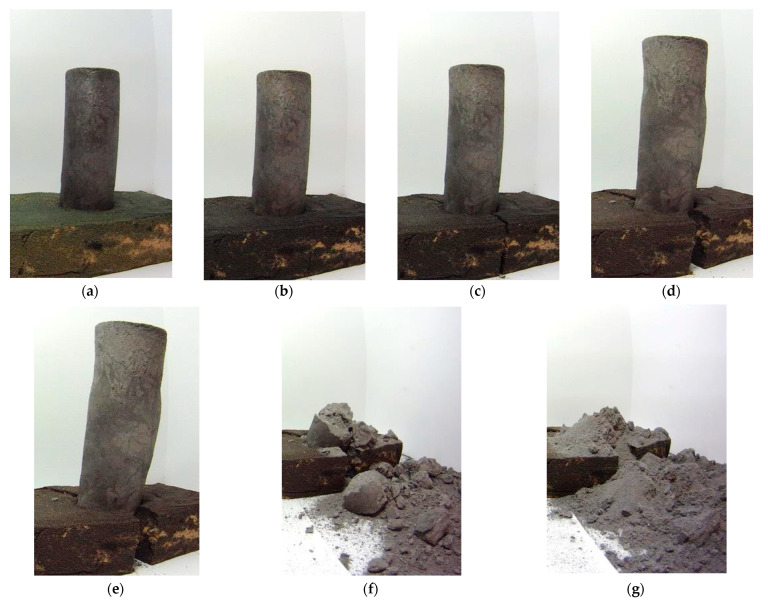
High-aluminum cast iron samples—photos taken after 1 day (**a**), 9 days (**b**), 12 days (**c**), 18 days (**d**), 20 days (**e**), 21 days (**f**), 31 days (**g**).

One of the samples was mounted in a specifically made bentonite mass stand and pictures were taken at intervals of 1 h. Time-lapse photos were taken with a GoPro digital camera, model YHDC5170 (San Mateo, CA, USA). Figure 3 shows the photos taken between days 1 and 21 of the study. Over a period of 21 days, the sample disintegrated.

When analyzing the photos, it could be noticed that the tested sample swelled and increased in volume over time. Therefore, it can be concluded that the disintegration of the material did not start with the surface layers, moving deeper into the material. These results indicate another cause of the spontaneous disintegration of the casting.

**Figure 4:**
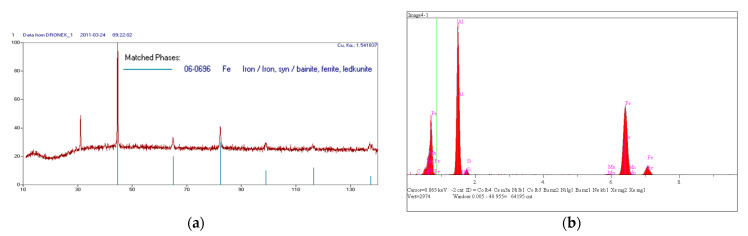
Decay powder diffractogram (**a**) and EDS X-ray microanalysis (**b**).

**Figure 5:**
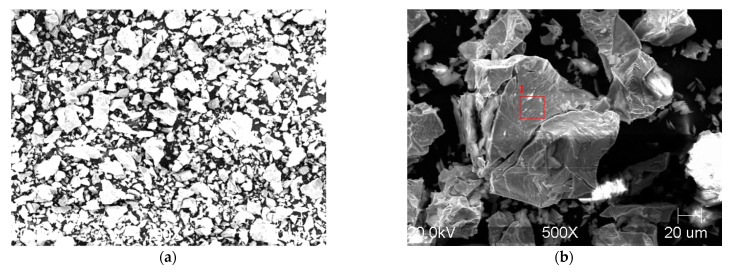
Microstructure of post-disintegration powder: different SEM microscope magnifications (**a**,**b**) and the site of EDS X-ray microanalysis (**b**).

In order to thoroughly investigate the possible causes of decay, the decay powder and the microstructure of high-aluminum cast iron were tested. The SIEMENS X-ray diffractometer, model Kristalloflex 4H (Munich, Germany), was used. A CuKα = 1.54 Å lamp was used. CuKα radiation, vertical goniometer and counter registration of the angle and position of the reflex were used. The measurement range of the 2-theta angle was 5–135°, step: 0.05°. The time of counting a single impulse was assumed to be 1 s. The voltage was 20 kV and the current 30 mA. The experimental data were processed in order to remove the background calculation of the position, integral intensity and peak height. For this purpose, the XRayan computer program was used (software version 421). Phase identification consisted of comparing the obtained signals with reference signals contained in the database (International Centre for Diffraction Data PDF-2, 2018). The results are shown in Figure 4a. In the presented image of the diffraction pattern of the decomposition powder, no undesirable metallic phases could be found. Figure 4b shows that the powder obtained during the self-destruction of the casting consisted of aluminum and iron oxides. The SEM microstructures of the resulting powder are shown in Figure 5. The samples were previously carbon-vapor-deposited to avoid static electricity. The exact chemical composition of measuring point 1 indicated in Figure 5 contains, by mass, 3.37% C, 3.18% O_2_, 37.50% Al, 1.85% Si, 0.01% S, 0.05% K, 0.45% Mn, 53.598% Fe. The EDS microanalysis is shown in Figure 4.

**Figure 6:**
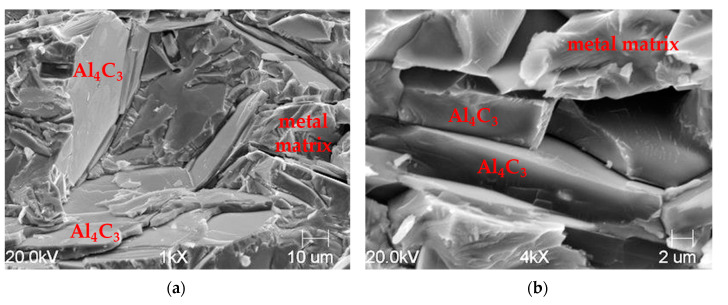
Sample fracture made of high-aluminum cast iron; SEM microstructure—magnification 1000× (**a**), 4000× (**b**).

**Figure 7:**
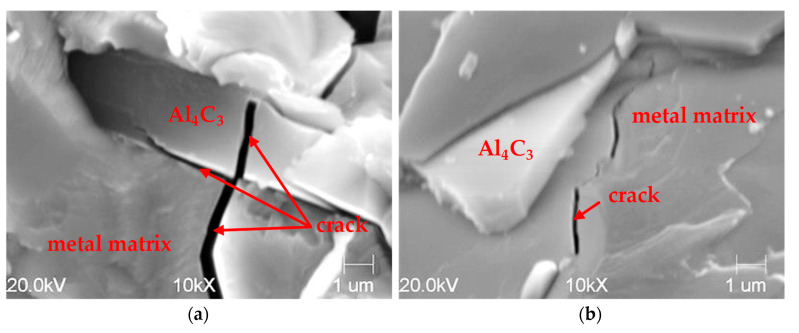
Sample fracture made of high-aluminum cast iron: visible cracks in carbide and metal matrix (**a**) and metal matrix (**b**) SEM microstructure.

The metallographic analysis of the fracture of the cast samples showed that the precipitation of the Al_4_C_3_ carbide was characterized by a lamellar morphology. These precipitations, with longitudinal and transverse dimensions in excess of 100 μm, had a thickness of 3–8 μm. The SEM microstructure of the fractures of the samples without and with a visible fracture is shown in Figure 6 and Figure 7.

In Figure 7, the fracture of the metal matrix and the Al_4_C_3_ carbide can be seen.

**Figure 8:**
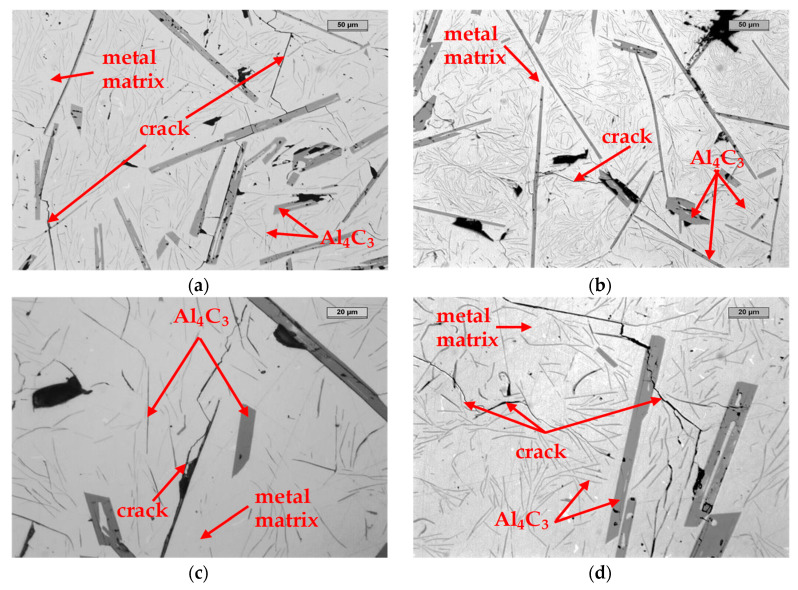
Microstructure of specimens of a high-aluminum cast iron with visible fractures of the metal matrix. Photos (**a**–**d**) present the structure of reference samples from high-aluminum cast iron, which were subjected to the phenomenon of spontaneous decomposition.

Figure 8 shows the precipitation of the Al_4_C_3_ carbide in the form of plates with a length exceeding 100 µm and a thickness of approximately 5–8 µm. These plates sometimes interpenetrated each other (Figure 8). Carbide dimensions of this size suggest the presence of high tensile stresses in the matrix during its contraction while cooling. These forces were large enough to destroy the Al_4_C_3_ carbides.

Figure 8 shows the matrix cracks along the carbide plates, which suggests that the mechanical strength of the carbide was higher compared to the metal matrix. During the crystallization of the alloy and its free contraction, aluminum carbide was able to resist the metal matrix, causing mechanical stress. The largest of them were accumulated on the periphery of the carbide, causing the notch effect, and hence a straight path to the destruction of the metal matrix. In an extreme case, when the bending strength R_g_ of the carbide was exceeded, it also failed, as shown in Figure 8. During the decohesion of the metallic matrix and the carbide, a micro-gap was formed, into which water vapor from the air could diffuse without any obstacles, thus reacting with the aluminum carbide in accordance with Reaction (1) described in the Introduction to this work.

Subsequently, Al_4_C_3_ began to increase its volume, causing the destruction of the casting in its entire volume, not only in the outer layers. After the formation of microcracks, water vapor could rapidly enter the internal structures of the casting, and, as a secondary reaction, hygroscopically interacting with the carbide, this could lead to its rapid destruction.

After analyzing the above observations, it seems advisable to use this type of treatment in order to prevent the decohesion of the metal matrix and Al_4_C_3_ carbide. Therefore, it can be assumed initially that the process of self-decay can be prevented in the following ways, among others:

- Reducing the size of the Al_4_C_3_ carbide precipitates to such values that the metal matrix, which shrinks during crystallization, is able to withstand the stresses caused by the Al_4_C_3_ carbide resisting it;
- Changing the shape of the carbide and reinforcing the strength of the metal matrix;
- Application of an appropriate heat treatment in order to change the shape of Al_4_C_3_ carbides.

In the next part of this publication, the above assumptions are considered. One further issue remains, i.e., the replacement of the Al_4_C_3_ carbide with another carbide with the use of carbide-forming elements, e.g., Ti, V, W. Such treatment may result in the complete elimination of the Al_4_C_3_ carbide by producing another with a changed shape. The compact shape of the new carbide, e.g., TiC, VC, WC, will not cause excessive stress during free shrinkage, contributing to the durability of the casting [5,11].

**Figure 9:**
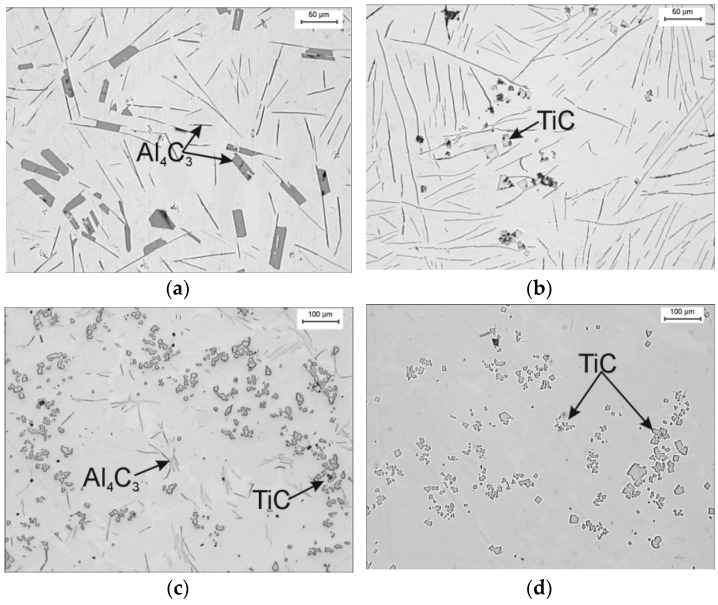
The microstructure of high-aluminum cast iron (**a**) and after addition of 1.3 %mass Ti (**b**), 2.7 %mass Ti (**c**), 5.4 %mass Ti (**d**); the structure subjected to spontaneous decomposition with different intensities (**a**–**c**), stable and durable structure (**d**).

The experiment shown in [5], which concerns the exchange of the Al_4_C_3_ carbide for a TiC carbide, proves that the hygroscopicity of the Al_4_C_3_ carbide and the prevention of Reaction (1) are crucial in terms of obtaining a material with good mechanical properties. Figure 9 shows the results of these studies.

Figure 9 shows the structure of a high-aluminum cast iron that underwent self-destruction (spontaneous decomposition) and after Ti was introduced into the cast iron in such an amount that led to the formation of a titanium carbide and the disappearance of the aluminum carbide. It was found that such a material is not subject to the self-destruction mechanism.

## 5. Reducing the Size of the Al_4_C_3_ Carbide Precipitates

**Figure 10:**
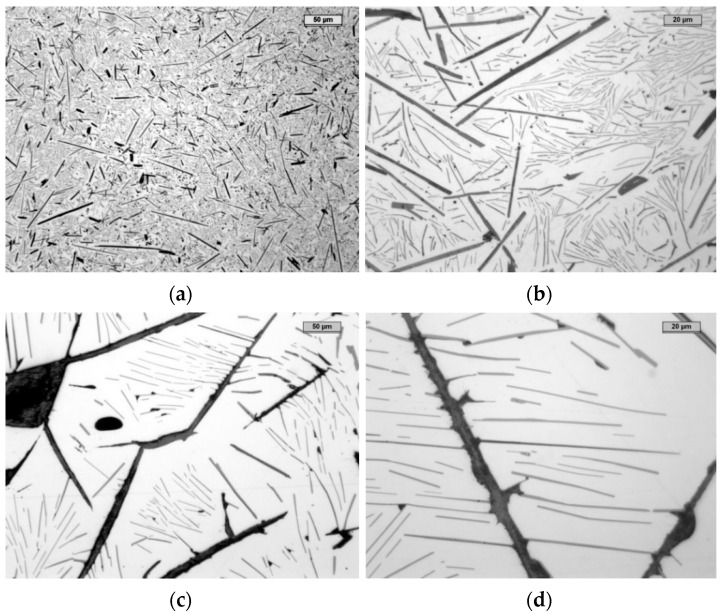
The microstructure of the reference sample cooled at 300 °C/s (**a**,**b**) and at 10 °C/h (**c**,**d**).

In line with the assumptions presented earlier, a special experiment was prepared. Two cast iron samples with the chemical composition specified in Table 1 were subjected to different crystallization times: relatively short, for the cooling rate of 300 °C/s (Figure 10a,b), and long, for the cooling rate of 10 °C/h (Figure 10c,d). It was assumed that, during such different extreme crystallization times, the precipitates of the primary Al_4_C_3_ carbide would have different dimensions.

For quick cooling, the first sample was poured into a mold made of copper, with the dimensions of the casting being ∅ = 45 mm in diameter and 4 mm thick, and the second into a sand mold with bentonite. The second cast in the form of a cylinder had the outer dimensions of diameter ∅ = 100 mm and height 100 mm. After the alloy was poured, the mold containing the still molten metal was placed in a temperature-controlled resistance furnace to ensure that it cooled to room temperature.

When analyzing the microstructures of both samples, it could be noticed that (as expected) the precipitation of the Al_4_C_3_ carbide in the case of the sample cooled at a high speed was relatively low. In the case of the sample cooled at a low speed, the Al_4_C_3_ precipitation was 5–10× greater compared to the first sample. The first sample, which cooled quickly, did not disintegrate, while the second sample, which cooled slowly in an oven, disintegrated within one week. This confirms the assumption that one of the reasons for the decomposition is the relatively large longitudinal size of the Al_4_C_3_ carbide, which, by inhibiting free shrinkage, resists the metal matrix, contributing to the decohesion of the matrix–Al_4_C_3_ carbide components.

It can be concluded that a change in the shape of the precipitation, which ultimately will not lead to the effect of the notch, will significantly affect the service life of this high-aluminum cast iron.

The practical use of the described anti-disintegration method is not easy to implement in the case of larger castings, but in the case of casting small details, e.g., in a water-cooled die, it is feasible.

## 6. Change in the Morphology of Al_4_C_3_ Carbide of Cast Iron with the Addition of Large Amounts of Chromium Precipitates and the Effect of Heat Treatment

Among the alloying additives used to improve the ductility of Fe-Al alloys, chromium deserves special mention. There are two possible explanations for its effect on enhancing ductility independent of the environment [13]. One of them is the theory that a Cr additive changes the arrangement of atoms and increases the spacing of screw superdislocations by decreasing the antiphase boundary (APB) energy. The second theory is that Cr addition can alter the surface condition of the sample and the oxidation mechanism, so that the adverse effects of the environment (hydrogen embrittlement) can be mitigated. Therefore, the above theory was used to produce a stable material that should not be subject to the process of spontaneous decomposition.

In the experiment, high-aluminum cast iron with the addition of chromium was melted. The chemical composition is presented in Table 2. During the melting process, chromium was added to the molten metal. Then, the metal was superheated to the temperature of 1510 °C, after which, at the temperature of 1420 °C, the samples were poured (for metallographic tests) into a metal die previously heated to 300 °C. The die made it possible to obtain four cylindrical samples with a diameter of 18 mm and a length of approx. 100 mm.

**Figure 11:**
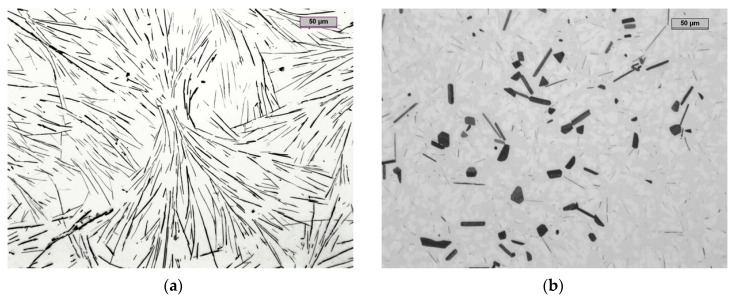
The microstructure of a sample made from high-aluminum cast iron with the addition of chromium, eutectic structure as cast (**a**) and hypereutectic structure after heat treatment (**b**).

Two of the four samples were additionally annealed in an oxidizing atmosphere furnace at 950 °C for 24 h and then cooled within the furnace. Then, metallographic specimens were taken from all samples (Figure 11). High-aluminum cast iron samples with the addition of chromium without heat treatment were subject to degradation over a longer period of time. Self-destruction started from one to two years after pouring.

**Figure 12:**
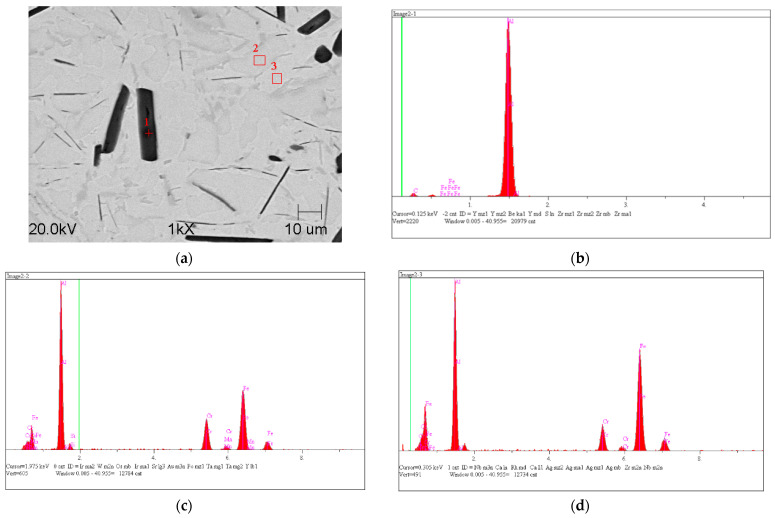
SEM microstructure with marked EDS chemical analysis points of a sample made from high-aluminum cast iron after heat treatment (**a**) and EDS X-ray microanalysis of carbide precipitation: No. 1 (**b**), No. 2 (**c**), No. 3 (**d**).

**Figure 13:**
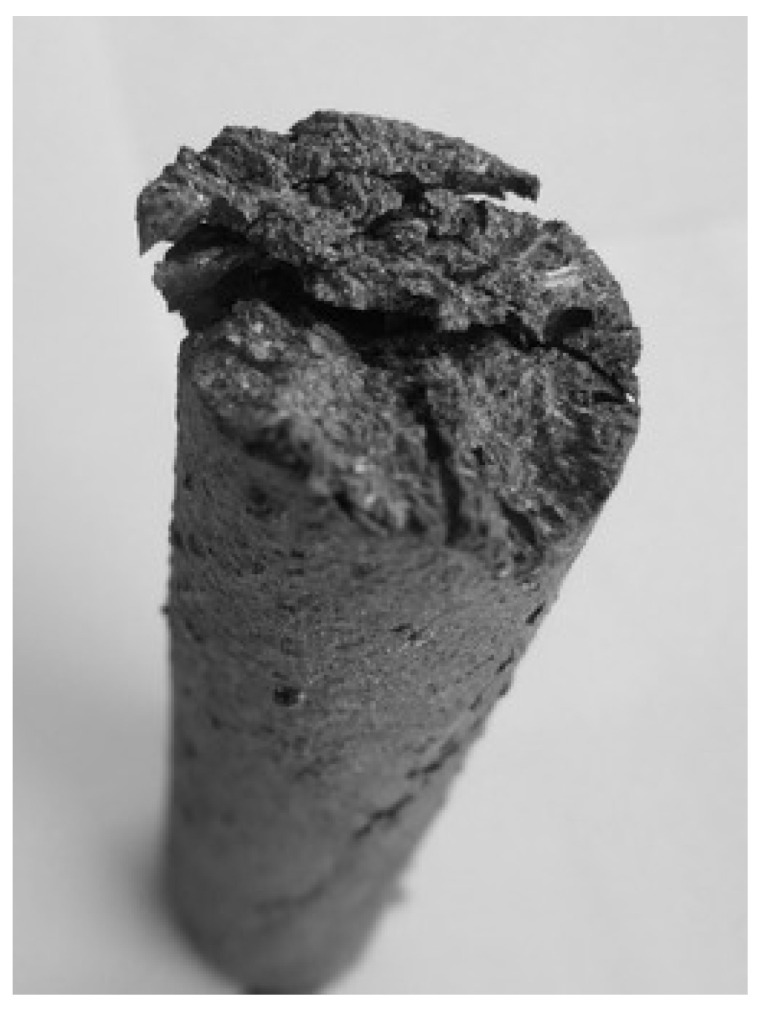
The sample without heat-treatment, one year after production—visible surface disintegration of structure.

The slender, long shapes of eutectic aluminum carbides, almost 100 μm long, took on different shapes and sizes after heat treatment. Cast iron obtained by combining aluminum and chromium obtained a structure composed of aluminum–chromium ferrite, reinforced with Al_4_C_3_ carbides. Thus, it had features that combined both chrome cast iron and high-aluminum cast iron. This type of cast iron is unknown in the literature. In other words, we obtained a usable high-aluminum cast iron with much higher chromium content, even exceeding 14%mass. The Al_4_C_3_ carbides after heat treatment are shown in Figure 11b and Figure 12a. The external appearance of the sample and its disintegration one year after its production are shown in Figure 13.

The metal matrix in the microstructure consisted of two chemically different phases composed of iron, aluminum and chromium. In order to determine the exact composition of the matrix and the visible aluminum carbides, a quantitative analysis of the chemical composition in the energy-dispersive X-ray (EDX) micro area was carried out.

The results of the quantitative analysis of the chemical composition of selected points are presented in Table 3, and the zones of their occurrence are presented in Figure 12b–d. This sample, despite the still existing aluminum carbide Al_4_C_3_, did not disintegrate and did not show the self-destruction mechanism.

The method developed and described above was patented and published in the patent description PL 222673 B1 [14].

## 7. The Effect of High-Aluminum Cast Iron Modification—A Minor Addition of Boron and Heat Treatment

A modification procedure was used to plasticize the metal matrix. For this, small amounts of boron and bismuth were used. The literature [15,16,17] shows that boron as a microadditive usually segregates along grain boundaries. For example, it can reduce the segregation of phosphorus at grain boundaries. Then, the hydrogen concentration at the grain boundaries decreases and thus the possibility of intergranular cracking is reduced. Boron segregation increases intergranular cohesion and strengthens grains boundary strength. Boron leads to low sensitivity to hydrogen-induced cracking and, generally, it is applied to improve ductility. Thus, it is an ideal microadditive to be used for castings made of high-aluminum cast iron, which has a tendency to self-destruct. In contrast, the micro-addition of Bi in high-quality grey iron leads to a change in graphite morphology [18]; in the case of high-aluminum cast iron, it was assumed that it would cause the fragmentation of Al_4_C_3_ precipitates. Thus, it was assumed that by adding these elements, the metal matrix would be strong enough not to be damaged by the action of the Al_4_C_3_ carbide plate.

The test melting of cast iron was carried out, the chemical composition of which is shown in Table 4.

**Figure 14:**
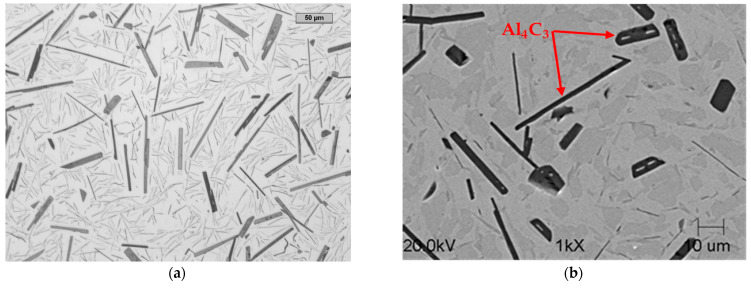
Microstructures of high-aluminum cast iron with the addition of boron and bismuth; hypereutectic structure as cast (**a**) and hypereutectic structure after heat treatment (**b**).

During the melting process, ferro-boron was added to the molten metal composed of high-aluminum cast iron. Then, a metal bath was superheated to a temperature of 1510 °C and, in the final stage, bismuth was added; at a temperature of 1420 °C, samples for metallographic tests were poured into a metal mold preheated to 300 °C. Four cylindrical samples with a diameter of 18 mm and a length of around 100 mm were obtained. Two of the samples were additionally annealed in an oxidizing atmosphere furnace at a temperature of 950 °C for 24 h, and then cooled within the furnace. The fracture for both sets of samples was fine-grained (Figure 14). The sample disintegrated after two months, which proved that the reinforcement of the metal matrix through the modification treatment extended the working time of the casting by 100% compared to the reference casting.

The precipitation of the Al_4_C_3_ carbide in the alloy was not eliminated, as can be seen in Figure 14. This is consistent with the assumption that such a small amount of boron could not completely replace this Al_4_C_3_ carbide with boron carbide. Figure 14 shows Al_4_C_3_ carbide precipitation in the form of plates with a length of approximately 50 μm. If a longer Al_4_C_3_ plate appeared, its thickness was much smaller than that shown in Figure 9 for the reference high-aluminum cast iron. Those carbides that exceeded a thickness of 5 μm were characterized by a length much less than 50 μm. This is key in the stable behavior of the structure of high-aluminum cast iron. After heat treatment, the alloy did not disintegrate. It is a relatively inexpensive casting alloy that may find application in industry. The method developed and described above was patented and published in the patent description PL 222671 B1 [19].

## 8. Summary

**Figure 15:**
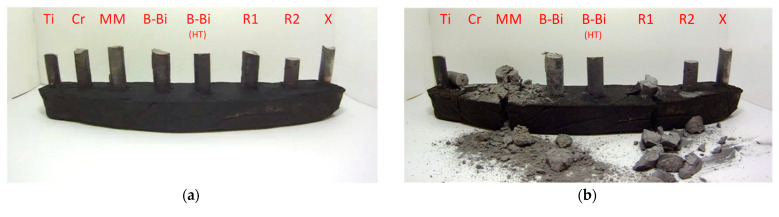

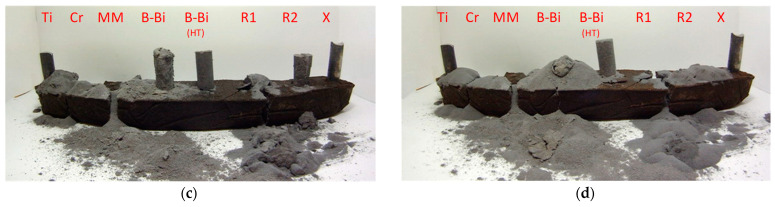
Cast samples of high-aluminum cast iron with addition of Ti—titanium, Cr—chromium, MM—rare earth elements, B–Bi—boron and bismuth, B–Bi (HT)—boron and bismuth after heat treatment, R1—reference sample (standard cooling rate), R2—reference sample (increased cooling rate) and X—know-how (an attempt will be made to implement into production); observation time: first day (**a**), 74 day (**b**), 148 day (**c**), 216 day (**d**).

To verify the research, several specimens were placed in a specifically made base and 21,000 images were taken for 7 months. Selected images from this set are shown in Figure 15. As can be seen, only three specimens withstood the test of time, i.e., high-aluminum cast iron with the addition of boron and bismuth and heat treatment (B–Bi (HT)), the sample with the addition of titanium (Ti), and the sample named know-how (X).

The research results demonstrate that the reaction of aluminum carbide Al_4_C_3_ with the environment is not the only cause of the mechanism of casting disintegration. These studies provide new data on the emerging stresses in the casting structure. This issue is related to the morphology of aluminum carbide Al_4_C_3_. It was found that one of the causes of casting breakdown is the relatively large longitudinal size of the Al_4_C_3_ carbide, which inhibits free shrinkage and resists the metal matrix by contributing to the decohesion of the metal matrix. The least expensive way to prevent this phenomenon is to modify the high-aluminum cast iron with boron and bismuth and carry out a dedicated heat treatment.

## 9. Conclusions

The article defines the mechanism of the spontaneous decay of high-aluminum cast iron castings, which is caused by the large relative size of Al_4_C_3_ carbide precipitates. The mechanism is as follows:

- During the crystallization of the alloy and its free shrinkage, aluminum carbide as a plate resists the metal matrix, causing mechanical stress accumulated mainly on the edges of the carbide, causing the notch effect and detachment of the carbide surface from the matrix. In an extreme case, when the bending strength R_g_ of the Al_4_C_3_ carbide is exceeded, it is also destroyed.
- During the resulting decohesion of the metal matrix and the Al_4_C_3_ carbide, a micro-gap is formed, into which water vapor contained in the air diffuses, thus reacting with the carbide.
- Subsequently, the precipitation of the Al_4_C_3_ carbide begins to increase its volume, causing the destruction of the casting in its entire volume, not only in the outer layers.
- After microcracks appear, water vapor enters the internal structures of the casting faster and, as a secondary factor, leads to its rapid destruction.

During this research, methods of counteracting the phenomenon of spontaneous decay were developed. The methods developed include:

- Increasing the speed of cooling. Thanks to this, the dimensions of the Al_4_C_3_ carbide precipitates are so small that the metal matrix, which shrinks during crystallization, is able to overcome the stresses caused by the Al_4_C_3_ carbide precipitation that resists it.
- The compact geometric shape of this carbide does not cause excessive stress during free shrinkage, contributing to the durability of the casting
- Changing the shape of the carbide precipitates and strengthening the strength of the metal matrix, e.g., by a modification treatment.
- Heat treatment of high-aluminum cast iron also contributes to changing the shape of carbide precipitates, which makes them durable.

## Figures and Tables

**Table 1:** Chemical composition of high-aluminum cast iron.

Element	C	Si	Mn	Al	S	Fe
**%mass**	0.91	0.29	0.25	34.70	0.01	remaining

**Table 2:** Chemical composition of high-aluminum cast iron.

Element	C	Cr	Si	Mn	Al	S	Fe
**%mass**	2.12	14.20	1.03	0.30	34.33	0.01	remaining

**Table 3:** Chemical composition of high-aluminum cast iron.

Element	C	Al	Cr	Fe	Mn	Si
	%Mass					
Phase No. 2	0.00	39.18	14.03	45.06	0.00	1.73
Phase No. 3	0.00	31.95	8.06	55.86	0.32	1.58
Carbide No.1	39.15	60.50	-	-	-	-

**Table 4:** Chemical composition of high-aluminum cast iron.

Element	C	Si	Mn	Al	S	Bi	B	Fe
**%mass**	2.08	1.08	0.5	36.02	0.01	0.05	0.11	remaining

## Data Availability

Not applicable.
